# Revolutionizing Maxillofacial Rehabilitation for Ocular Defects: The Impact of Three-Dimensional Printing and Sublimation Transfer Technique on Changing Horizons

**DOI:** 10.7759/cureus.49706

**Published:** 2023-11-30

**Authors:** Ankita Pathak, Mithilesh M Dhamande, Seema Sathe, Smruti Gujjelwar, Sheetal R Khubchandani

**Affiliations:** 1 Prosthodontics, Sharad Pawar Dental College and Hospital, Datta Meghe Institute of Higher Education and Research, Wardha, IND

**Keywords:** three dimensional printing, sublimation transfer technique, ocular defect, prosthetic eye, ocular prosthesis

## Abstract

An absence or any disfigurement to the eye leads to psychological handicap for the patient. An ocular defect is a defect of an eye because of a cyst, road traffic accident, or enucleation of an eye due to infection. While correcting this type of defect, maxillofacial prosthodontists should consider all aspects such as esthetics, comfort, and functions of the ocular prosthesis, which gives a lifelike appearance to the prosthesis. A prosthetic eye wearer patient complained of asymmetry and opaque appearance of the ocular prosthesis. The patient had lost his eye in a road traffic accident and has been wearing a prosthesis for 8-12 months but is not pleased with how the prosthesis looks. A novel prosthesis created by the use of sublimation transfer technology and three-dimensional printing to improve the esthetics exactly replicates the contralateral normal eye. In a proposed case report, an algorithm for the fabrication of customized ocular prostheses was improved. A smooth blend of conventional as well as digital methods is used to optimize the results.

## Introduction

Physical and psychological disturbances due to the loss of an eye can cause significant mental and emotional handicaps to the patient. Rehabilitation of ocular defects in such unfortunate patients reduces psychosocial distress and boosts the patient’s confidence level [1,2]. Loss of an eye or an unfortunate absence of an eye can be caused due to trauma, congenital defect, or cancer-induced enucleation of the eye. Beauty is a greater recommendation than any letter of introduction and the eye is not only an organ of vision but also the epitome of facial expression, and cosmetic appearance to the patient as well. However, rehabilitating with an artificial or prosthetic eye restores the mental trauma and self-esteem of the patient [3].

Optimum results with enhanced esthetics appear along with symmetry and replicating the exact colour, and contour of the present eye in the prosthetic eye. Because asymmetry might cause squinting, accurate alignment of the iris disc in the scleral wax pattern is critical for creating the custom-made artificial eye [3]. Prefabricated eye shells or custom-made artificial eyes can be used to rehabilitate this type of defect. Because the iris should be bilaterally symmetrical, precise positioning of the custom-made iris disc on the scleral wax pattern is essential. This case report describes the rehabilitation of ocular defects by replacing older prostheses with new ones by combining conventional as well as digital methods to modify the position, colour, and contour of the iris by using the sublimation transfer technique [4].

## Case presentation

A 58-year-old Asian male patient, a farmer, visited the maxillofacial prosthodontics department. The patient complained of disfigurement and an asymmetrical appearance due to the loss of his right eye. After recording the past medical history, it was revealed that the patient was asymptomatic two years ago. He had met with an accident, and subsequent infection had led to the enucleation of the right eye. The patient underwent rehabilitation of the right eye and had been wearing a prosthesis for two years. However, the patient was not satisfied with the prosthesis due to the displaced position of the iris and a squinted eye appearance.

**Figure 1:**
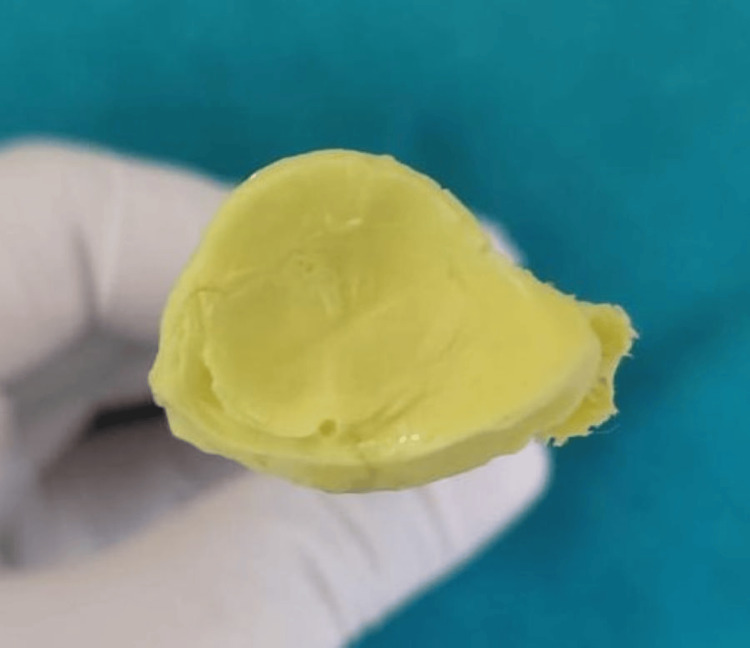
Preliminary impression with irreversible hydrocolloid material Image credit: Ankita Pathak

**Figure 2:**
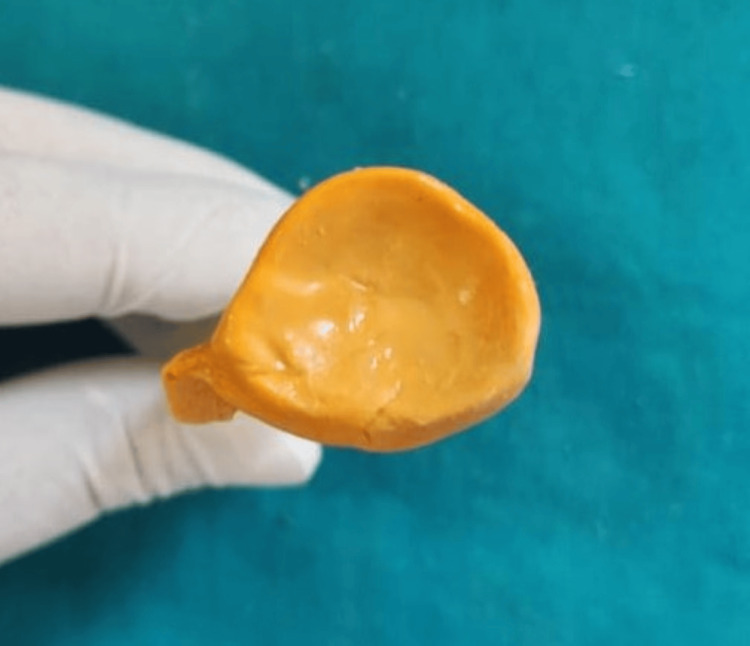
Final impression recorded with light body polyvinyl siloxane material Image credit: Ankita Pathak

Using a novel approach, a treatment plan was devised to fabricate a customized eye prosthesis. The preliminary impression was made with irreversible hydrocolloid, and the final impression was recorded with light-body polyvinyl siloxane material as shown in Figure 1 and Figure 2.

**Figure 3:**
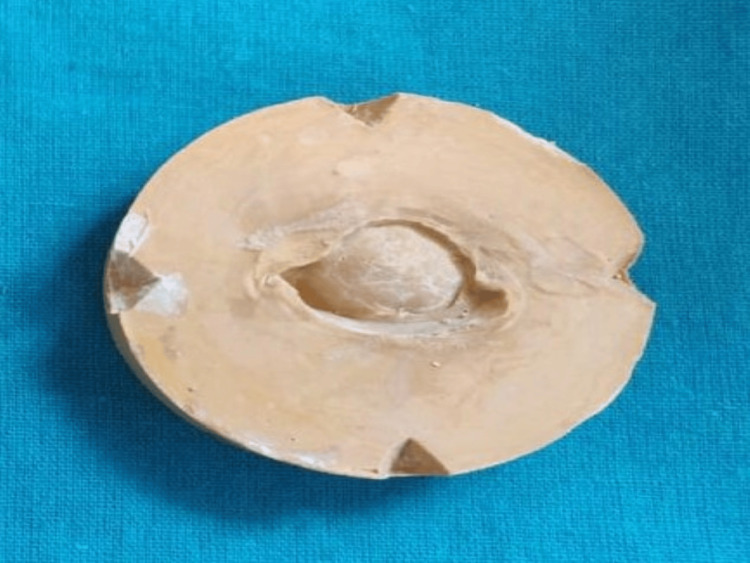
Fabrication of cast with die stone material Image credit: Ankita Pathak

The cast was then fabricated using type 4 dental stone or die stone as the die stone records all the minute details of the impression, illustrated in Figure 3.

**Figure 4:**
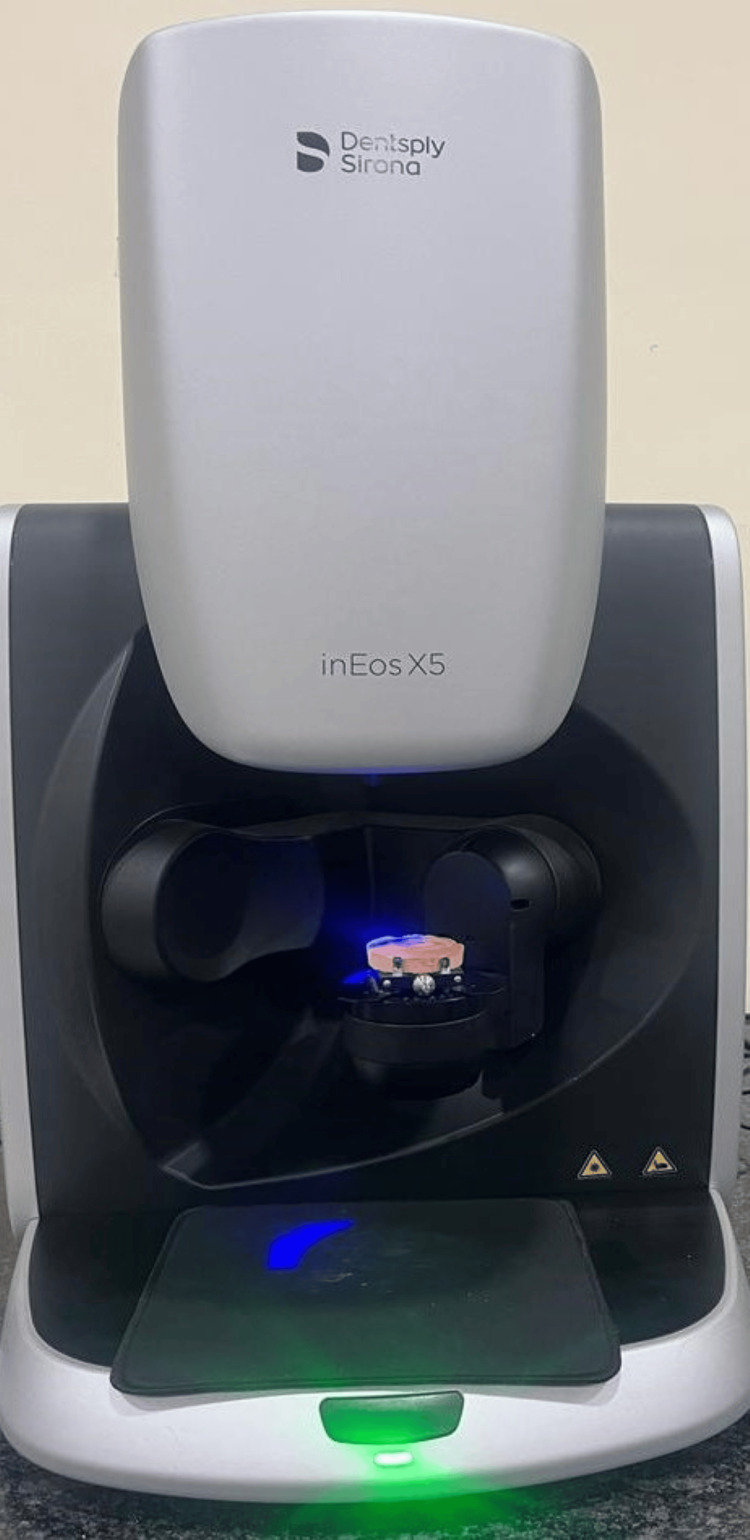
Scanner inEOS X5 used to scan the fabricated cast Image credit: Ankita Pathak

**Figure 5:**
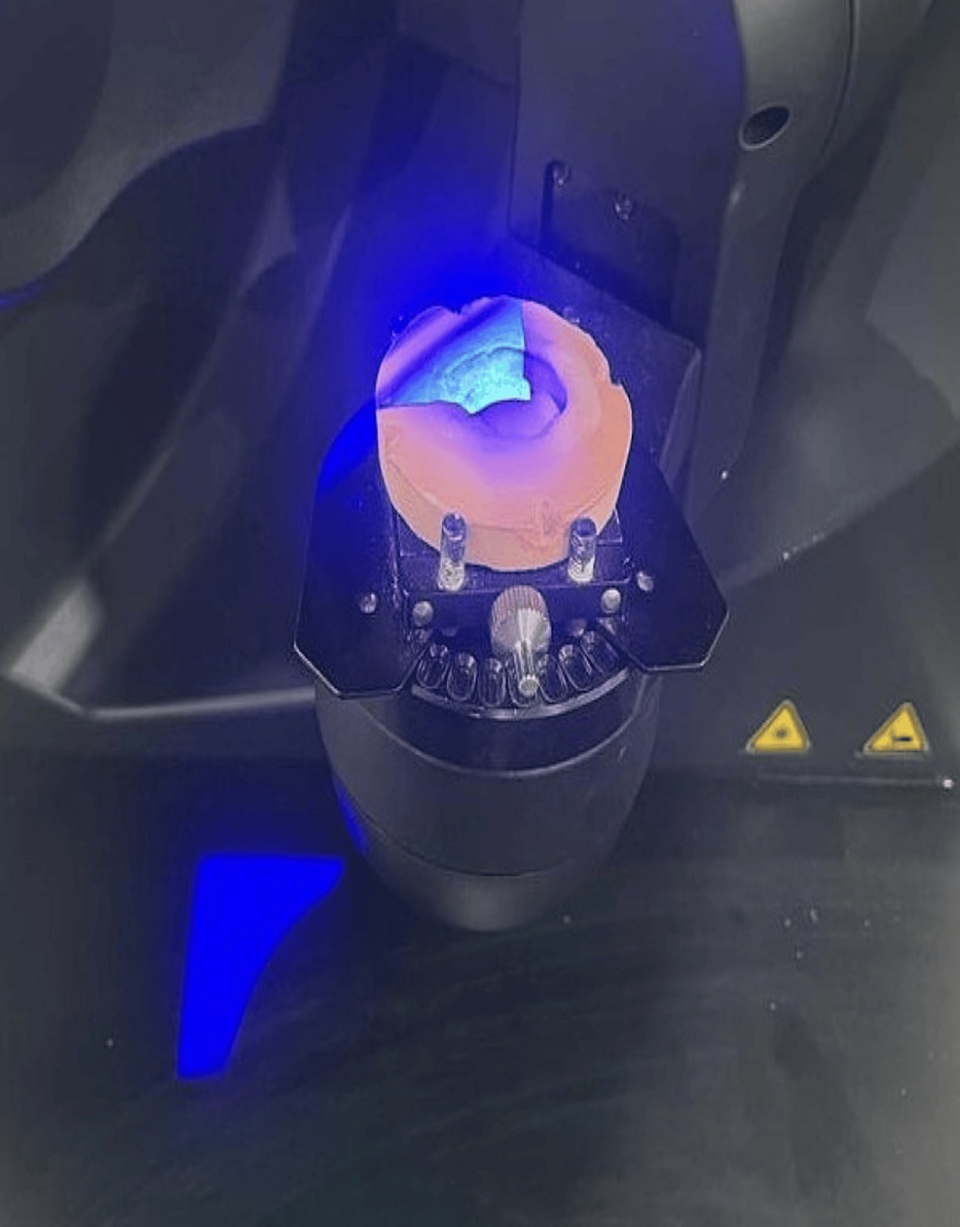
Scanning of the cast with inEOS X5 scanner Image credit: Ankita Pathak

After cast fabrication, the cast was scanned using computer-aided designing (CAD) software (exocad 5xt software, Darmstadt, Germany) with an inEOS X5 scanner (Dentsply Sirona, Charlotte, USA) used to scan the cast, as depicted in Figure 4 and Figure 5.

**Figure 6:**
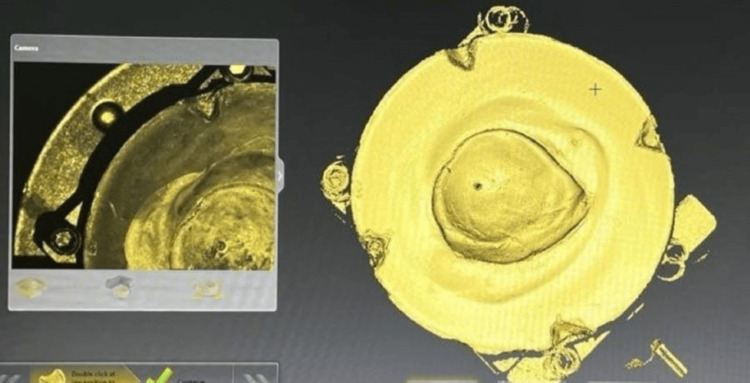
A scanned model of the fabricated cast Image credit: Ankita Pathak

**Figure 7:**
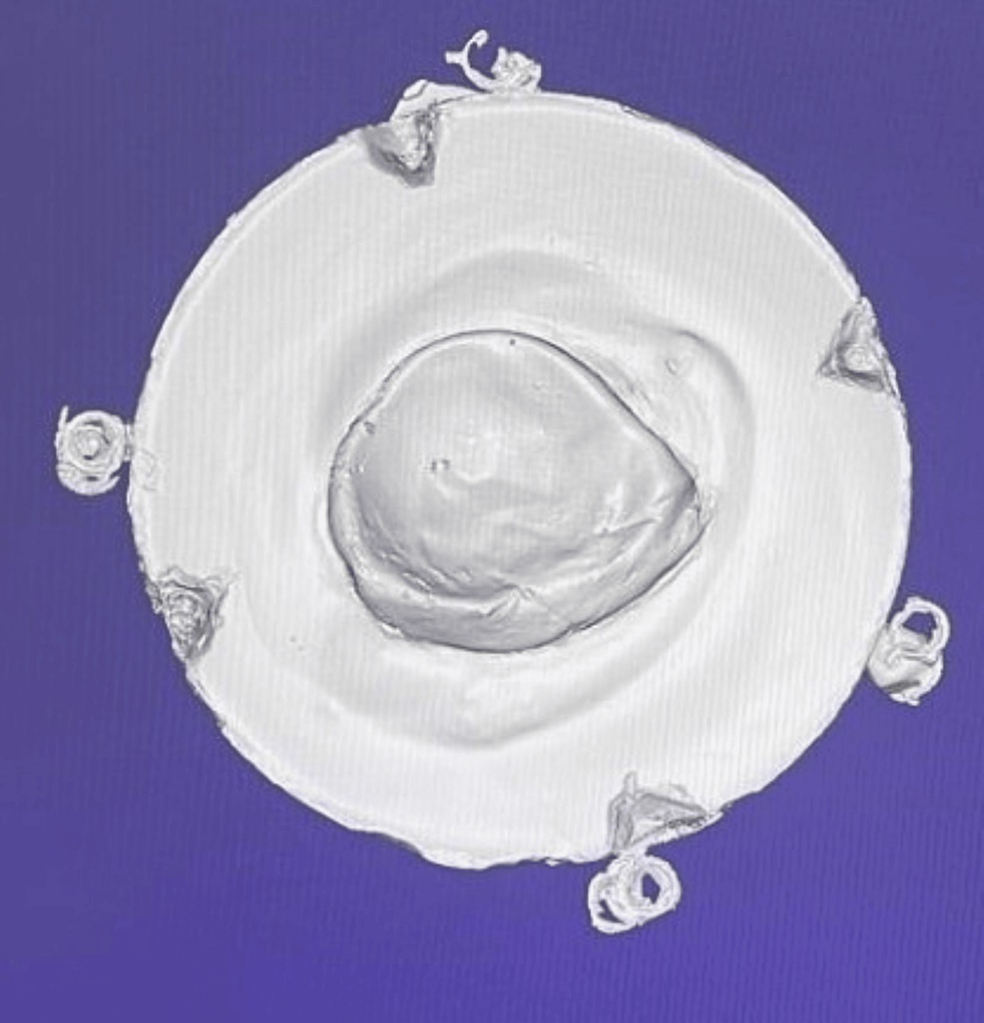
Removal of artefacts from the scanned model Image credit: Ankita Pathak

Scanned images were produced at different angles from the model, and artefacts on the scanned model were removed, which were visualized on CAD software, as shown in Figure 6 and Figure 7.

**Figure 8:**
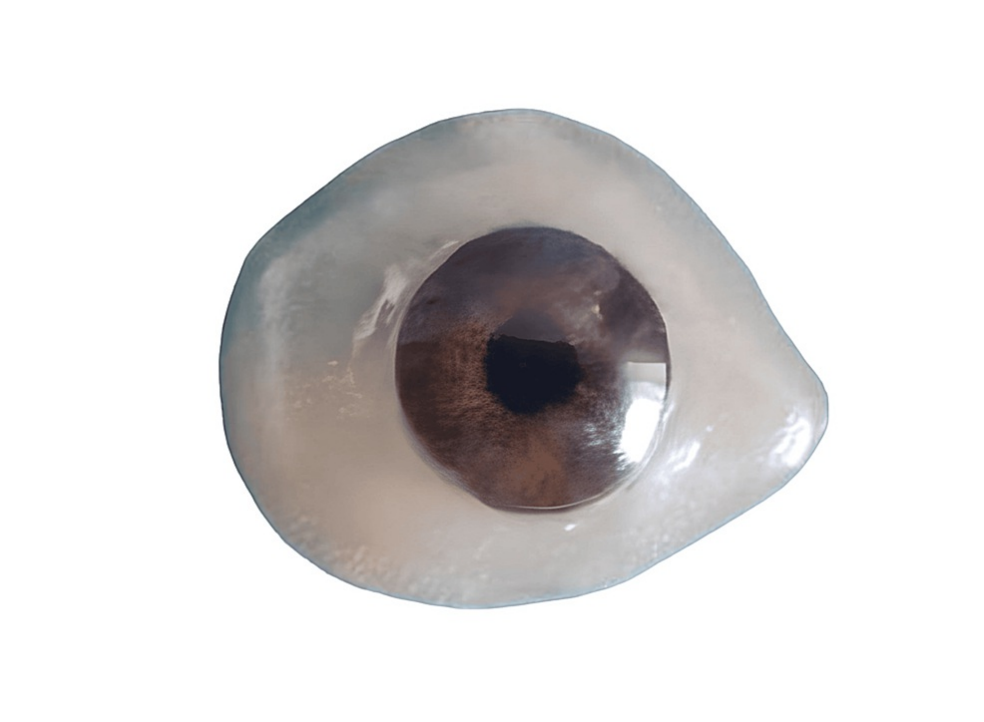
Sclera and iris printed on transfer paper Image credit: Ankita Pathak

The 3-dimensional (3D) model data is converted into a stereolithographic (STL) file and stored. A 3D printer is employed to print the required model. Various colours are available from the library, and the closest matching colour to the sclera is selected. Following the installation of the 3D printer, a slicing file containing the entire 3D model data is produced. In the 3D printer, this file is uploaded, and the prosthesis is printed. To acquire graphical data of the iris and blood vessels, the contralateral normal eye of the patient was captured through a digital camera (Canon 450D, Canon Inc., Tokyo, Japan) at a distance of around one foot. Employing a mirroring tool, the image was duplicated, and subsequent adjustments were made to the blood vessels while maintaining a constant iris size of 11 mm. Subsequently, utilizing Adobe Photoshop CS4 (Adobe Systems Inc., San Jose, USA), specific regions of the image containing the pupil, iris, and blood vessels were meticulously delineated. The brightness and contrast parameters of the image were then systematically manipulated to generate a calibrated image file suitable for printing. For printing the iris, the sublimation transfer technique is utilized. A specially crafted frame, designed to conform to the curved shape of the ocular prosthesis, is placed onto the sublimation transfer device. Subsequently, the 3D-printed ocular prosthesis is positioned within this frame. The printed transfer paper is then inverted, and heat is applied following the vacuuming of the transfer paper and the 3D-printed prosthesis into contact. Once the sublimation ink has been successfully transferred and adhered to the 3D-printed product, the transfer paper is removed. The image is then printed on transfer paper, as depicted in Figure 8.

**Figure 9:**
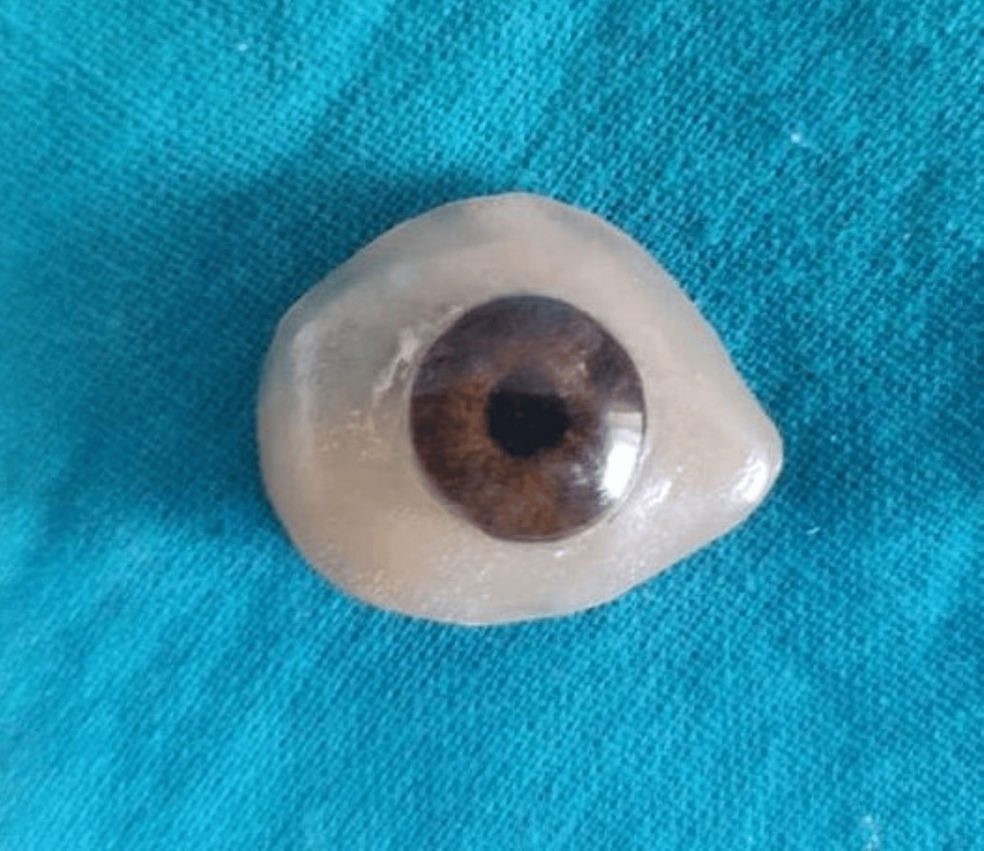
Designed ocular prosthesis printed by 3D printer Image credit: Ankita Pathak

Through the application of the sublimation transfer technique, the inked portion was vacuumed and securely adhered to the 3D-printed eye shell or ocular prosthesis (3D STAR-6 S, Diofun Inc., Gunpo, Republic of Korea). The designed ocular prosthesis was produced using a sublimation transfer process specifically optimized for curved surface printing, as depicted in Figure 9.

**Figure 10:**
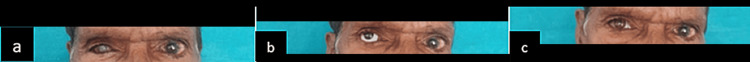
(a) Remaining scleral tissue in the right eye, (b) older prosthesis in situ, and (c) prosthesis fabricated by sublimation transfer technique Image credit: Ankita Pathak

Following the complete fabrication of the prosthesis, it was inserted. The iris positioning, colour, contour, and centralization of the iris were greatly appreciated compared to the older prosthesis, as depicted in Figure 10.

After the prosthesis was inserted, the patient performed a range of movements to detect any sharp areas or projections. Subsequently, any identified problems were addressed through additional finishing and polishing. Post-insertion instructions were then provided to the patient, including the recommendation to remove the prosthesis at night. The patient received guidance on maintaining the hygiene of the prosthesis by cleaning it with mild soapy water and was instructed to store the prosthesis in water when not in use. Subsequent follow-up visits were scheduled for the patient, with the first occurring after three days. It is noteworthy that the patient expressed satisfaction with the aesthetics, functionality, and comfort of the prosthesis.

## Discussion

The conventional technique of ocular prosthesis is time-consuming, tedious, labour-intensive, and involves multiple manual processes. Although commercially available prefabricated eye shells can be used, custom-made, patient-specific designed ocular prostheses offer ultimate aesthetic results [4,5]. The sublimation transfer technique is a semi-automated method that integrates both conventional and digital approaches smoothly. In comparison to the fabrication of a conventional eye prosthesis, which takes around ten hours, the suggested technique takes roughly eight hours [5].

Various methods for printing on curved surfaces have been developed, including dye sublimation transfer printing, laser printing, direct inkjet printing, and laser printing [6-8]. The dye sublimation transfer technique was chosen for this process as the ocular prosthesis exhibited noticeable curvature [9-11]. This method ensures no tearing or peeling of the design, making it suitable for printing on cotton fabrics and curved-surfaced items [12,13].

3D printing, also known as additive manufacturing (AM) or rapid prototyping (RP), is a promising technique that utilizes a layer-by-layer fabrication approach to produce items [14]. 3D printing technology has been effectively employed in scaffold-based tissue engineering for the production of complex 3D scaffolds using both direct and indirect procedures [15]. Many processes in the production of prostheses are still artisanal and time-consuming [16,17]. Modern ocular prosthesis manufacturing techniques, including 3D printing and digital imaging, can shorten treatment times, better reproduce patient features, eliminate the need for facial imprints, and reduce the complexity of wax pattern sculpting [18-20].

The fundamental limitation of the suggested approach is that skill and expertise are required during the fabrication of such prostheses. Also, the required cost is slightly expensive as compared to conventional techniques. However, production time is significantly reduced, and technical expertise is no longer necessary, as a semi-automated process replaces the majority of the manufacturing process. Furthermore, this approach requires expertise and resources to conduct 3D graphics and modelling that were previously unavailable [4].

Therefore, this clinical study introduces a unique semi-automated process for constructing customized ocular prostheses that reduces the time and expertise required. The ability to save 3D modelling data is particularly useful, as the data can be utilized if the patient's ocular prosthesis is lost or damaged. The suggested method facilitates patients' access to superior, personalized ocular prostheses, thereby enhancing their quality of life [4].

## Conclusions

The digital era has extended its influence into maxillofacial prosthodontics, particularly in the realm of eye prostheses, where the correct positioning of the iris is crucial for success. To achieve an accurate replica of the contralateral eye in terms of contour and colour, the integration of 3D printing and advanced techniques, such as the dye sublimation transfer method, is essential. The proposed clinical report demonstrates that the colour, contour, and shape of the eye can be precisely replicated using this technique.
